# Enhancing the mechanical properties and surface morphology of individualized Ti-mesh fabricated through additive manufacturing for the treatment of alveolar bone defects

**DOI:** 10.3389/fbioe.2023.1284359

**Published:** 2023-11-06

**Authors:** Lingxu Wang, Fangfang Wang, Saimi Ayisen, Tianshui Ren, Xiaoping Luo, Penglai Wang

**Affiliations:** ^1^ School of Stomatology, Xuzhou Medical University, Xuzhou, China; ^2^ School of Stomatology, Nanjing University, Nanjing, China

**Keywords:** additive manufacturing, titanium meshes, heat treatment, mechanical properties, synergistic finishing technology of electric field and flow field (EFSF), surface characteristics, bacterial adhesion

## Abstract

Titanium meshes are widely utilized in alveolar bone augmentation, and this study aims to enhance the properties of titanium meshes through heat treatment (HT) and the synergistic finishing technology of electric field and flow field (EFSF). Our findings illustrate that the titanium mesh exhibits improved mechanical properties following HT treatment. The innovative EFSF technique, in combination with HT, has a substantial impact on improving the surface properties of titanium meshes. HT initiates grain fusion and reduces surface pores, resulting in enhanced tensile and elongation properties. EFSF further enhances these improvements by significantly reducing surface roughness and eliminating adhered titanium powder, a byproduct of selective laser melting printing. Increased hydrophilicity and surface-free energy are achieved after EFSF treatment. Notably, the EFSF-treated titanium mesh exhibits reduced bacterial adhesion and is non-toxic to osteoblast proliferation. These advancements increase its suitability for clinical alveolar bone augmentation.

## 1 Introduction

Titanium meshes are widely used for repairing complex bone defects and have shown good bone augmentation effects (Merli et al., 2015 Boyne et al., 1985). When using commercial meshes, adapting to each patient’s complicated atrophic bone structure takes considerable time and efforts, increasing the operation time and the risk of infection (Sumida et al., 2015). Compared with to prefabricated titanium meshes, additive manufacturing enables the production of customized three-dimensional titanium meshes. These meshes meet the individual needs of patients through preoperative design and digital virtual reconstruction. This approach facilitates precise bone grafting procedures, streamlines surgical processes, and shortens operation time, presenting promising clinical applications (Sumida et al., 2015).

Currently, titanium alloys such as Ti-6Al-4V (TC4) are commonly used for titanium mesh fabrication. However, the leakage of aluminum and vanadium from TC4 into the complex oral environment raises concerns about their potential harm to human bodies (Gomes et al., 2011; Rao et al., 1996). On the other hand, pure titanium offers several advantages. It is a single-element material that is lightweight with high specific strength, low thermal conductivity, minimal magnetism, x-ray translucency, excellent corrosion resistance, and favorable biological properties (Dong et al., 2020). However, pure titanium exhibits lower mechanical strength compared with titanium alloys.

In surgery, titanium meshes need to be sufficiently rigid to withstand pressure from the overlying flap, muscle movements, and chewing loads (Rakhmatia et al., 2013). Studies have suggested that an adult male individual can generate biting forces ranging from 45 to 68 kg (441.3–666.8 MPa), indicating that the bending fracture of the titanium mesh should be tested within this range (De Angelis et al., 2021). However, in the case of titanium mesh implantation, the surgical area typically does not directly bear the full force of occlusal pressures. A study suggests that stress from intraoral soft tissues can be the primary cause of displacement in bone grafting materials, leading to inadequate bone regeneration (Buser et al., 1996). Therefore, a testing pressure of 21N, which was intended to simulate the maximum force that healthy intraoral soft tissues could exert (Ulrich Sommer et al., 2014), was used to assess the mechanical performance of the titanium mesh (Lee et al., 2017) This approach provided a more realistic scenario of the conditions that the mesh was likely to encounter in clinical practice.

Selective laser melting (SLM) is a prevalent method in additive manufacturing. During the rapid cooling phase of the SLM process, significant temperature gradients are formed within the metal specimens, potentially causing deformation or curling. A proposed solution to these structural defects is HT, which has been successful in mitigating residual stresses and enhancing both the mechanical and biological properties of SLM-manufactured titanium meshes (Wang et al., 2020; Scarano et al., 2018; , 2020; Li et al., 2021). Studies indicate that HT refines grains, enhances fusion, increases the elongation rate, and improves the mechanical strength of the specimens (Wang et al., 2020; Li et al., 2021). However, to date, data on the strength characteristics of pure titanium meshes are lacking.

Furthermore, the additive manufacturing of titanium meshes results in the adherence of unmelted metal powder to the surfaces, making the surfaces highly rough and difficult to polish. The rough surfaces not only stimulate gingival attachment but can also contribute to bone adhesion, making retrieval more challenging. Once the titanium meshes are exposed to oral cavities, the rough surfaces provide favorable conditions for bacterial adhesion. Researches have shown that increased surface roughness enlarges the surface area of the materials; also, features such as pits, grooves, scratches, and cracks on rough surfaces can influence bacterial adhesion and serve as favorable sites for biofilm formation (Ribeiro et al.,2012; Santhosh Kumar et al., 2019). Highly roughened materials increase the likelihood of bacterial adherence, increasing wound healing duration and postoperative infection (Dank et al., 2019).

Various methodologies are currently employed for modifying the surface of titanium mesh, including mechanical abrasion, electrolytic polishing, sandblasting, computer numerical control machining, chemical mechanical polishing, and laser polishing (Wang et al., 2016). Notably, electrolytic polishing, in particular, is commonly employed to reduce the roughness of titanium implants produced through additive manufacturing. However, using corrosive chemicals in this approach raises concerns regarding environmental impact and safety. Electrochemical plasma polishing (ECPP) (combining conventional electrolysis with atmospheric plasma processes) has been used for removing contaminants and reducing oxide layers to address these issues (Yang et al., 2016). This synergistic technique produces exceptionally smooth and glossy surfaces with enhanced corrosion resistance. ECPP is an environmentally friendly and cost-effective approach, making it a promising choice for reducing the roughness of 3D-printed titanium implants (Zeidler et al., 2016). However, researches on ECPP for titanium mesh substrates are still limited.

This study aimed to optimize the mechanical and surface properties of additively manufactured pure titanium meshes using two methods: vacuum HT and a novel synergistic finishing technology called synergistic finishing technology of electric field and flow field (EFSF). The study evaluated the bending resistance, elongation rate, tensile strength, biocompatibility, and bacterial adhesion of the additively manufactured pure titanium meshes (before/after vacuum HT and with/without EFSF), thus providing experimental data for exploring the more promising titanium meshes.

## 2 Materials and methods

### 2.1 Preparation of experimental specimens

**FIGURE 1:**
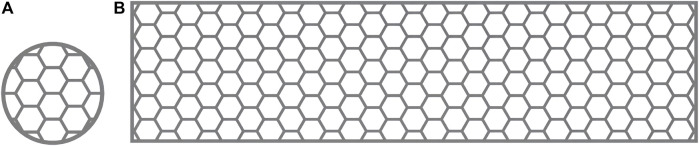
**(A)** Biological test specimen and **(B)** mechanical performance test specimen.

Commercially pure titanium (cpTi), which is used in current dental implants (McCracken, 1999), can be classified into four grades based on purity and oxygen content (Liu et al., 2017). These grades exhibit variations in corrosion resistance, ductility, and strength. This study selected flow-treated TA1 titanium powder (15–53 μm; Jiangsu Vilory Advanced Materials Technology Co., Ltd., China) (Table 1) as the powder material. Modeling and slicing were performed using Magics 22.0 software (Materialise, Belgium), and the Tr150 printer (Nanjing Qianzhi Intelligent Technology Co., Ltd., China) was employed for layer-by-layer laser printing under an argon atmosphere. Tables 2, 3, respectively, list the printing and HT parameters for As-built laser melting deposition parts. The printing parameters were set as follows: laser intensity of 120 W, scanning speed of 1,000 mm/s, and layer thickness of 0.030 mm. Wire cutting and support removal were conducted after printing, followed by sandblasting treatment (110-μm alumina sandblasting agent; Renfert, Germany), to refine the surface. HT was conducted in a vacuum HT furnace (RZF110014230S; Shanghai Refan High-Temperature Equipment Co., Ltd., China). Biological test specimens were prepared as circular disks with a diameter of 10 mm and a thickness of 0.3 mm (Figure 1A), as well as hexagonal mesh structures with a diameter of 2 mm (Figure 1). After ultrasonic cleaning (KQ-250DE; Kunshan Ultrasonic Instruments Co., Ltd., Suzhou, China) with deionized water, the specimens were soaked in ethanol for 3 h and then dried for further use.

**TABLE 1:** Chemical composition of the TA1 alloy powder.

Element	Ratio (%)
Ti	Bal
Fe	0.0021
C	0.0011
H	0.0049
O	0.012
N	0.002

**TABLE 2:** SLM titanium printing parameters.

Printing parameter	Laser intensity (W)	Scanning speed	Hatch space (µm)	Layer thickness (µm)
	120	1,000 mm/s	80	30

**TABLE 3:** HT parameters.

HT	Heating rate	Temperature (°C)	Hold time (h)	Method of cooling
	8°C/min	700	1.5	Air cooling

**FIGURE 2:**
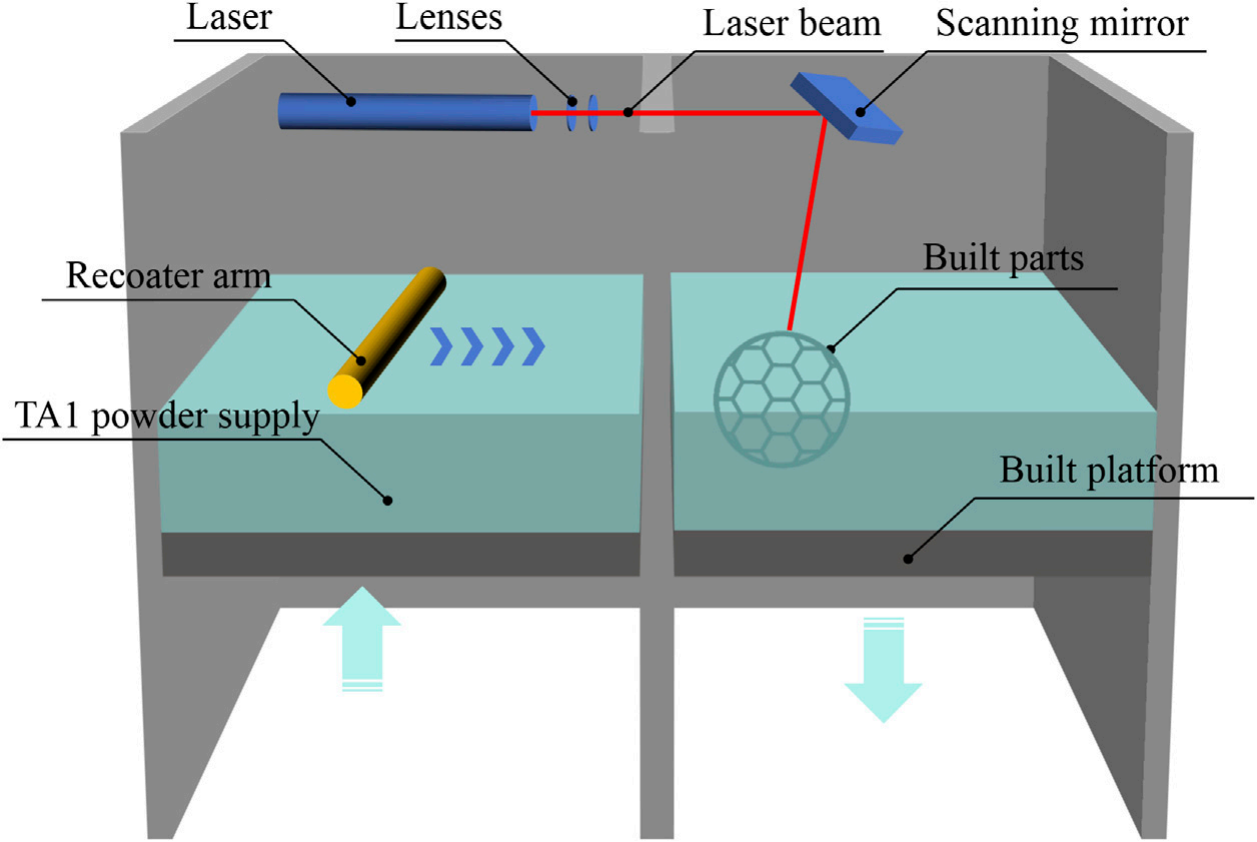
SLM printing process flowchart.

According to the requirements of YB/T5349-2014 for the test specimens for evaluating mechanical performance, a rectangular mesh thin-plate specimen with a length of 60 mm, a width of 15 mm, and a thickness of 0.3 mm was designed using 3-matic digital software. The mesh configuration was hexagonal (Figure 1B). The designed sample was saved in ". stl” format. The design file of the 0.3-mm sample was sent to a 3D printer, and the medical-grade pure titanium TA1 powder was used to print the mechanical performance test sample 2 of the titanium mesh. The relevant HT temperature and printing parameters were based on a previous study (Li et al., 2021) and the pilot experimental results. We processed the biological and mechanical performance test samples by printing and HT based on the requirements listed in Table 1. The SLM printing process flowchart for all specimens is depicted in Figure 2.

The mechanical performance test specimens were categorized into two groups: the As-built group and the heat-treated HT group. The specimens for biological testing and surface performance testing were categorized into the As-built, HT, and EFSF groups. The specimens in the EFSF group underwent surface treatment, and the samples were sequentially washed with an ultrasonic cleaner (90 kHz, 90 W output power), ethanol, and deionized water. The plasma polishing equipment (SFM20D-T; Nanjing Additive Manufacturing Research Institute Development Co., Ltd., China) was used, and an appropriate amount of electrolyte (2 wt% aqueous solution, pH = 5–7) was prepared in the working tank, with the electrolyte preheated to 55°C. The cathode tool and anode specimen were connected to the cathode and anode of the power supply system, respectively. The anode specimen was fixed in the working tank, rotating at 50 rpm centered on the workpiece axis. The micro-nano bubble generation device was activated, delivering an electrolyte containing micro-nano bubbles to the appropriate position on the surface of the anode specimen. The flow rate of this device was 0.6 m³/h, and the gas dissolution rate was 10%. The plasma polishing process was initiated by activating the constant-voltage power supply set at 280 V, operating a frequency of 500 Hz, pulse width of 100 s, voltage of 350 V, current of 2.5 A, and a duration of 10 min. The workpiece was removed, cleaned, and dried after polish.

### 2.2 Experimental methods

#### 2.2.1 Surface crystallographic observation

The As-built group and HT group samples were polished using standard metallographic procedures and etched with a corrosive solution (2 mL of HF + 4 mL of HNO3 + 94 mL of H2O). The microstructures were observed using a laser scanning confocal microscope (LSM800; Zeiss, Germany).

#### 2.2.2 Mechanical testing

In the tensile test, the tensile strength and fracture elongation were measured for specimens in both the HT and the As-built groups. The test was conducted using a universal testing machine (Autograph DCS-10T; Shimadzu, Kyoto, Japan) at a crosshead speed of 1.0 mm/min. The tensile test was conducted using a video extensometer with a strain rate of 1 × 10³ s−^1^ at room temperature. The tensile performance of the samples was derived from the average of seven independent measurements for each group. The yield strength (σys) was calculated using the 2% offset method.

In the three-point bending test, each group contained seven samples. Three-point bending tests were performed using a computer-controlled electronic universal testing machine (MTS; MTS Systems, MN, USA) and following the requirements of ISO 7438:2020. At an ambient temperature of 28°C, a force at a rate of 1 mm/min was applied vertically on the centerline of the samples. Constant vertical pressure was applied until the specimen was fractured, and the maximum bending or breaking force was recorded. The bending strength was calculated from the linear elastic area of the stress−strain curve. The calculation formula was as follows:

$$\sigma =\frac{3FL}{2bh^{2}}$$

where σ represents the bending strength (MPa), F represents the maximum bending force (N), L represents the beam span (mm), b represents the sample width (mm), and h represents the sample height (mm). Seven specimens were tested for each group, and the data were presented as mean ± standard deviation.

For the As-built and HT groups, Vickers hardness (WHW Microcre Optics-Mech; Shanghai Yanrun Optomechanical Technology Co., Ltd., China) was assessed on five specimens from each group. The measurements were conducted using a 9.8-kg load and a dwell time of 10 s at five distinct locations per specimen. Vickers hardness indentations appeared as diamond-shaped marks with a central "+" symbol. The Vickers hardness was calculated by measuring the lengths of the two extended diagonals of this symbol.

#### 2.2.3 Surface characterization

The internal scaffold and outer edge of the polished specimens were observed under a stereo microscope at a magnification of 100 times. The surface roughness of the three groups of titanium specimens was measured using a JB-4C precision surface roughness tester (Shanghai Taiming Optical Instrument Co., Ltd., Shanghai, China). Five specimens were randomly selected from each group, and three measurement points were taken on each specimen. The parameters of average roughness (Ra) and root mean square deviation (Rz) were determined. Ra corresponded to the arithmetic mean of the absolute values of the deviations of the profiles of a given sample length. Rz corresponded to the sum of the maximum peak height and the maximum valley depth within the sampling length. The water contact angle of the samples was measured at room temperature using a contact angle meter (JC 2000D2A; Shanghai Zhongchen, China) with a droplet volume of 0.013 mL, and three measurements were taken and averaged. The contact angle was measured with two different liquids, water and diiodomethane, to calculate the surface free energy (SFE). The SFE was calculated using the Owens and Wendt equation (Kasemo and Gold, 1999):

$$\gamma_{L}∙\left(1+\cos\theta \right)=2∙\left({\left(\gamma_{L}^{d}∙\gamma_{S}^{d}\right)}^{\frac{1}{2}}+{\left(\gamma_{L}^{p}∙\gamma_{S}^{p}\right)}^{\frac{1}{2}}\right)$$

#### 2.2.4 Bacterial culture and adhesion

Further, 1 mL of *Staphylococcus aureus* (Sa) 8325-4 was placed in a test tube, and 5 mL of Luria–Bertani (LB) culture (containing 1% glucose) medium (without antibiotics) was added to it. The tube was then placed on a shaker overnight (37°C, 250 rpm, 12 h) and stored at 4°C for later use. The bacterial suspension density was adjusted to 1 × 109 CFU/mL using a serial dilution and plate counting method with 1 μL of the bacterial suspension. LB culture medium was added to the liquid until the final density reached 1 × 106 CFU/mL. The metal samples were divided into three groups: As-built group, HT228group, and heat-treated followed by EFSF group. Each group contained 8 specimens andwas further divided into a 2-h group and a 4-h group. They were placed in 24-well culture plates and sterilized with gamma radiation (25 kGy), and 10 mL of the 1 × 106 CFU/mL liquid was added to each well. After 2 and 4 h, scanning electron microscopy (SEM) and fluorescence laser confocal microscopy were performed to observe bacterial adhesion.

#### 2.2.5 SEM observation of bacteria on different surfaces

After rinsing the samples with 0.01M phosphate-buffered saline (PBS) to remove surface-floating bacteria, the samples were transferred to six-well culture plates and 2.5% glutaraldehyde (AGAR, Stansted, UK) was added. They were then stored at 4°C overnight and washed with 0.01M PBS (twice, each time for 10 min). The samples were dehydrated with 50%, 70%, 90% ethanol, and absolute ethanol, followed by conversion with isoamyl acetate. Bacterial adhesion was observed under a Hitachi S-4800 (Hitachi, Tokyo, Japan) field emission SEM operated in secondary electron detection mode.

#### 2.2.6 Bacterial adhesion and fluorescence microscopy counting

The samples were washed with PBS (0.01M) twice to remove loosely attached or unattached bacteria on the specimen surfaces. They were then stained with the fluorescence oxidation–reduction dye Syto 9 at 37°C for 30 min, avoiding exposure to light. The bacterial observation was performed using fluorescence laser confocal microscopy within a 1.5 × 1.5 mm^2^ field of view. After colony formation, the number of bacteria was counted in 10 random areas of each substrate using Image-Pro software. Data were statistically analyzed using one-way analysis of variance (ANOVA) with Tukey’s test, and the experiment was repeated three times.

#### 2.2.7 Cell viability testing

MC3T3-E1 mouse osteoblast cells (National Collection of Authenticated Cell Cultures, China) were cultured in a humidified atmosphere at 37°C with 5% CO2. The medium used consisted of 10% fetal bovine serum and 1% penicillin/streptomycin for cell viability testing. The pretreated As-built, HT, and EFSF samples were placed in 48-well plates and immersed in 200 μL of fresh culture medium. Then, 500 μL of a cell suspension with a density of 1.0 × 104 cells/mL was evenly seeded onto the surface of each sample. Five parallel samples were prepared for each group (*n* = 5). All samples were placed at the bottom of the 48-well plate and immersed in 200 μL of fresh culture medium. Further, 500 μL of medium containing MC3T3-E1 cells was added to each well. The samples were divided into control and experimental groups. Additionally, 1 mL of PBS was added to the unused wells. After intervals of 1, 4, and 7 days, the culture medium was replaced with a mixed solution of 700 μL of the medium and CCK-8 solution in a 10:1 ratio. After incubating at 37°C for 2 h, 100 μL of the solution was transferred from each well to a 96-well plate, and the absorbance at 450 nm was measured using an enzyme-linked immunosorbent assay (ELISA; Sunrise-basic Tecan, Austria) reader. The cell viability was calculated as follows:

$$Cell\;Viability\left(\%\right)=OD450\left(sample\right)/OD450\left(control\right)\times 100\%$$

Toxic grade assessment was based on the six-grade toxicity rating standard (G.X. Pei et al., 2006), as listed in Table 4.

**TABLE 4:** Relative growth rate and toxicity grade.

Relative growth rate	Toxicity grade	Evaluation results
≥100	0	Qualified
75–99	1	Qualified
50–74	2	Re-review
25–49	3	Failed
1–24	4	Failed
0	5	Failed

### 2.2.8 Statistical analysis

The statistical analysis was performed using IBM SPSS Statistics version 22.0 (IBM, NY, USA). A one-way ANOVA in conjunction with Tukey's post hoc test was employed for the statistical analysis. Each experiment was conducted in triplicate and repeated three times to ensure robustness. All results were presented as mean values accompanied by their respective standard deviations. A comparative t test was carried out at a confidence level of 95% to assess the significance of differences among the various groups. Specifically, P values <0.05 indicated significant differences.

## 3 Results

### 3.1 Metallographic observation and mechanical property testing

**FIGURE 3:**
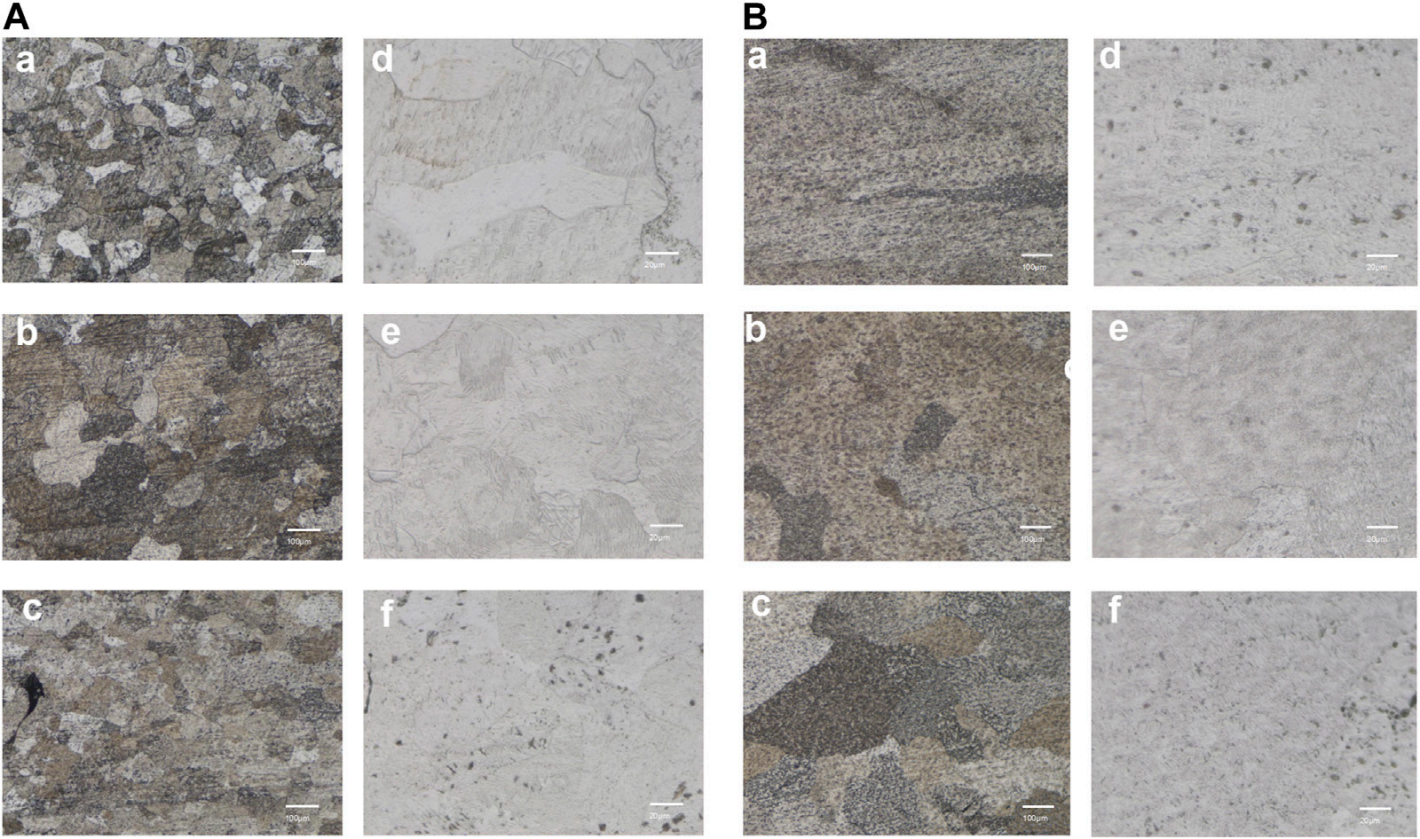
Microstructure of the SLM-printed TA1 samples (representing results of the **(A)** As-built **(B)** HT, **(a–c)** are the 100x metallographic micrographs at printing angles of 0°, 45°, and 90° respectively; **(d–f)** are the corresponding 500x laser confocal images.

The metallographic structure morphology of the As-built samples at 0°, 45°, and 90° is depicted in Figure 3. The emergence of *ß* columnar crystals was observed following direct printing of the alloy, with a width of 70–100 μm and height exceeding 1,000 μm. High-power laser confocal microscopy indicated the formation of a large number of α′ martensite needles, which were 0.5–2 μm wide. Additionally, black defect tissues were scattered throughout the structure, which were likely surface pores with incomplete crystal fusion.

A significant fusion of grains occurred following the HT treatment (700°C for 90 min). The higher heating temperature in HT compared with the non-heat-treated state led to an increase in the size of the grains after fusion. The observation of high-power backscatter images revealed the presence of some residual α′ martensite structures within the heat-treated sample. The number of surface pores significantly reduced, and the grain boundary of the HT sample was relatively clean. The number of circular pores, which are a type of printing defect, reduced in size to 20–30 μm. Further fusion of the unfused refined grains was observed over an extended time.

**FIGURE 4:**
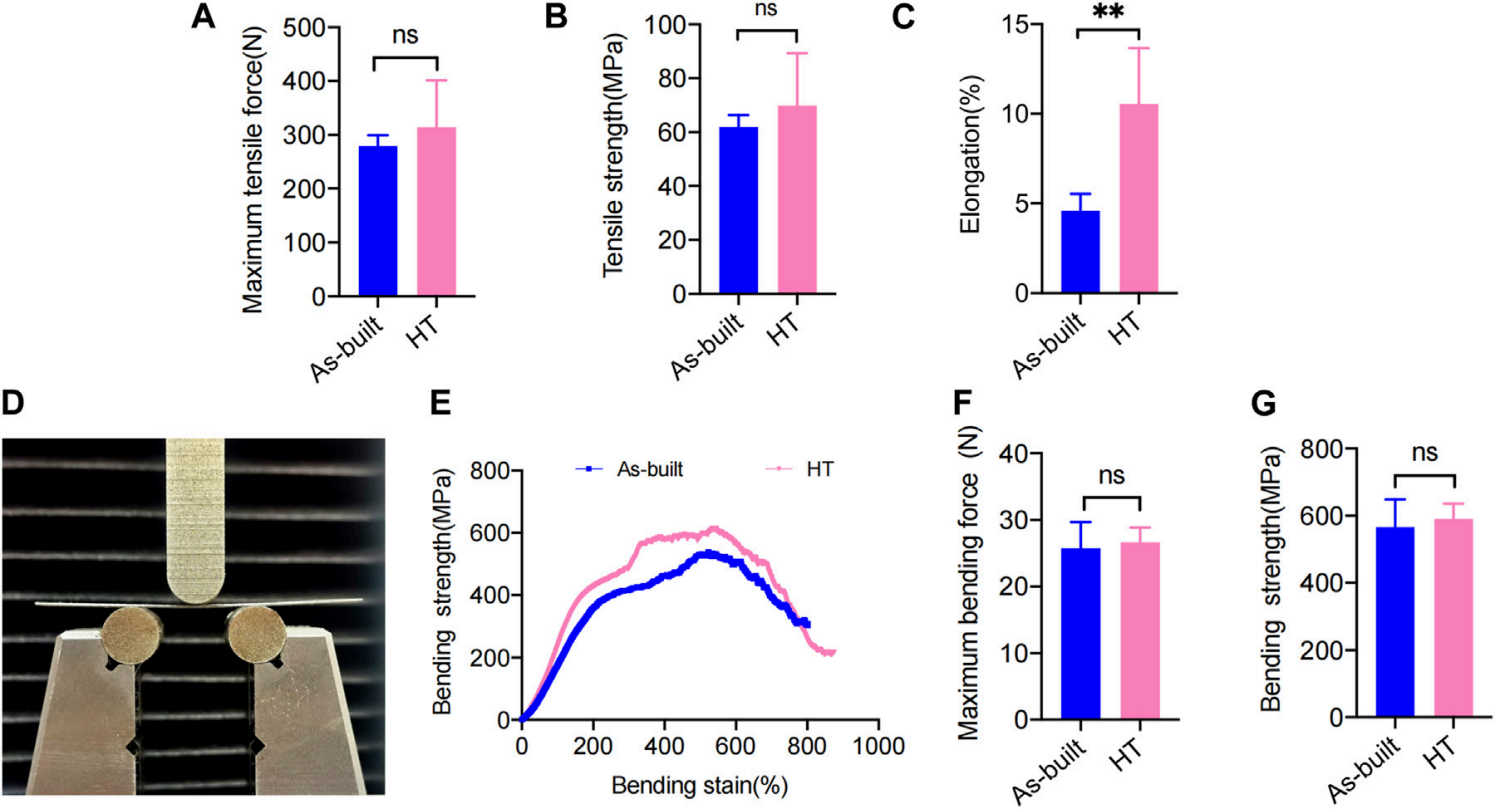
Statistical analysis of the mechanical test. **(A)** Maximum tensile force. **(B)** Tensile strength. **(C)** Elongation. **(D)** Front view of the bending test. **(E)** Stress−strain curves. **(F)** Maximum bending force. **(G)** Bending strength (*p < 0.05; **p < 0.01; ***p < 0.001).

The mechanical property assessment involved tensile tests performed for the As-built and HT samples. The As-built titanium mesh showed an average maximum tensile force of 297.3 ± 19.95 N and a tensile strength of 61.83 ± 4.53 MPa (Figures 4A, B). The HT samples demonstrated an increased maximum tensile force (314.0 ± 87.35 N) and tensile strength (69.83 ± 19.50 MPa) compared with the As-built samples, indicating a trend toward enhanced tensile strength with the HT protocol, despite the lack of statistical significance (*p* > 0.05).

In terms of elongation, the HT samples (10.56% ± 3.11%) showed a significant increase compared with the As-built samples (4.59% ± 0.93%) (Figure 4C), reaching statistical significance (*p* < 0.001). The As-built titanium mesh exhibited a maximum bending force of 21.63 ± 6.936 N and a bending strength of 566.2 ± 82.90 MPa in the three-point bending tests. A slight increase was observed in the HT samples, showing a maximum bending force (26.65 ± 2.20 N) (Figure 4F) and an increase in bending strength (590.9 ± 45.36 MPa) (Figure 4G) compared with the As-built samples, despite no statistical significance (*p* > 0.05).

### 3.2 Vickers hardness testing

**FIGURE 5:**
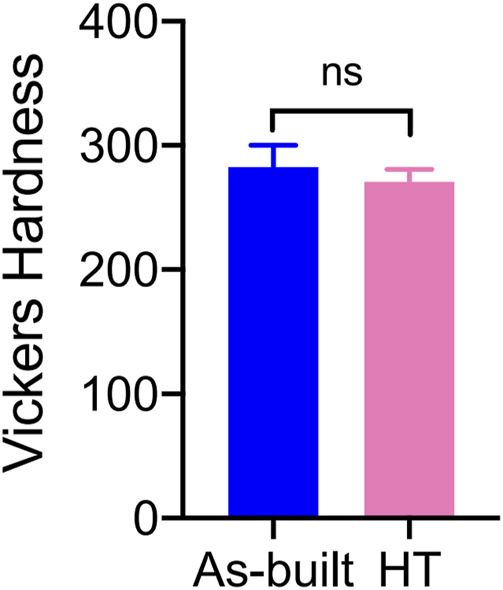
Comparison of surface Vickers hardness between As-built and HT titanium meshes.

The Vickers hardness values are shown in Figure 5. The Vickers hardness value for the HT group was 270.8 ± 10.16 HV, whereas the Vickers hardness value for the As-built group was 282.9 ± 17.11 HV. The difference between the two groups was not statistically significant (*t* = 1.920, *p* = 0.0708).

### 3.3 Analysis of surface morphology of EFSF-treated titanium mesh

**FIGURE 6:**
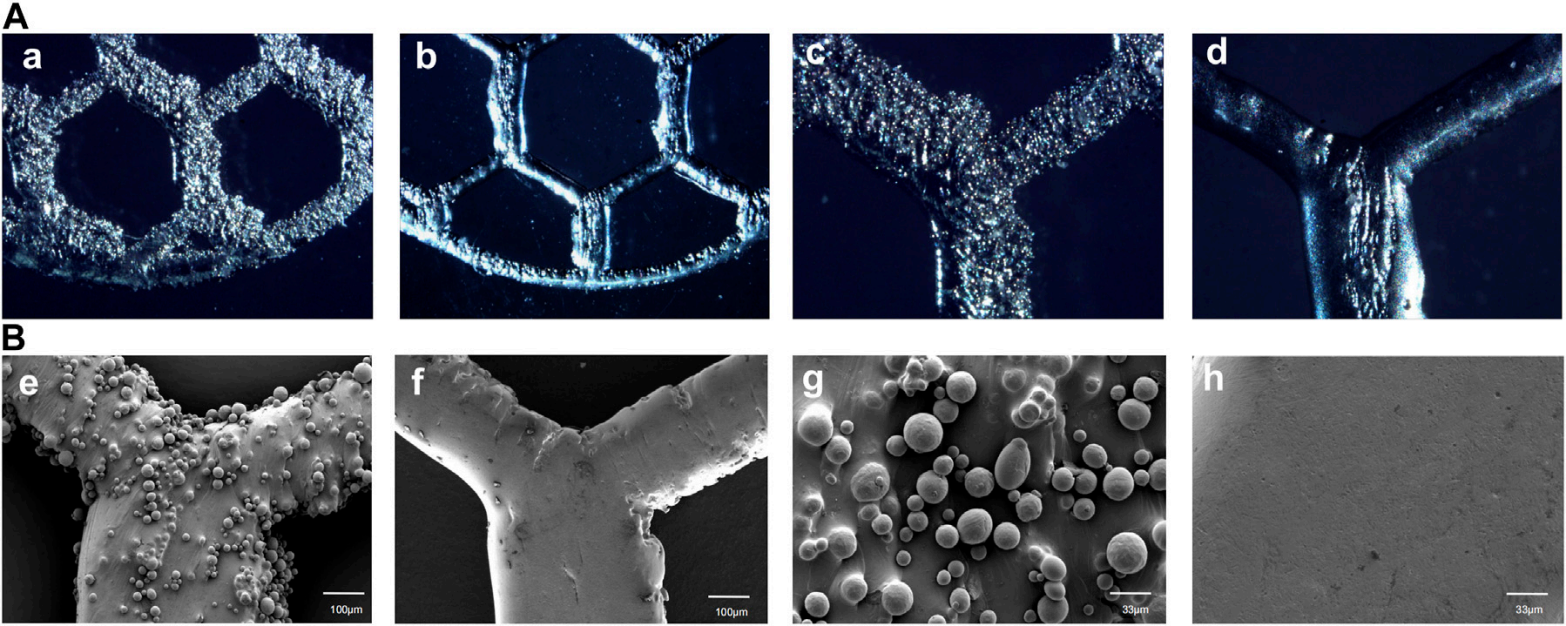
High-magnification optical microscope images of As-built characterization and EFSF-treated surfaces at ×50 magnification **(a, b)** and at ×100 magnification **(c, d)**, as well as SEM images of As-built and EFSF-treated surfaces at ×100 magnification **(e, f)** and at ×300 magnification **(g, h)**.

The optical microscope and SEM images of the specimens before and after EFSF treatment are shown in Figure 6. After SLM printing, a significant amount of residual titanium powder accumulated on the surface of the printed parts, resulting in surface roughness (Figures 6A, C, E, G). The inner side of the titanium mesh scaffold exhibited the highest powder accumulation, making it challenging to remove the adhered titanium powder manually; the powder tended to aggregate along the printing direction. A relatively smooth polished surface was obtained after EFSF treatment, with most of the adhered titanium powder removed (Figures 6B, D, F, H). However, larger defects on the surface could not be polished away, and potential cracks were present.

**FIGURE 7:**
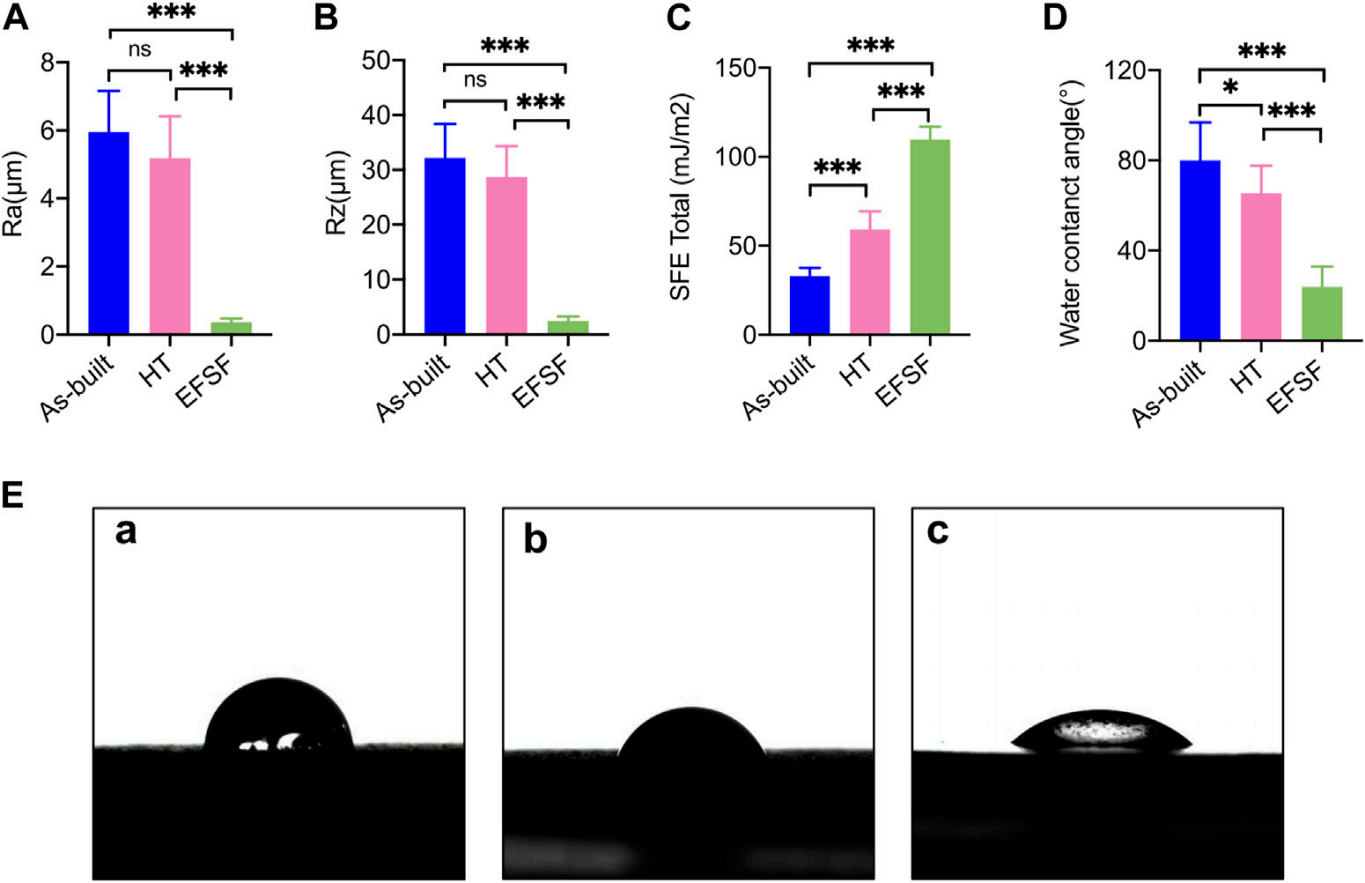
Roughness parameters **(A)** Ra and **(B)** Rz, SFE values **(C)**, and water contact angle values **(D)** of the specimens under different treatment conditions. **(E) (a)** Photograph of water droplet shape on the As-built scaffold after printing, **(b)** on the scaffold after HT, and **(c)** on the scaffold surface after EFSF (*p < 0.05; **p < 0.01; ***p < 0.001).

Figures 7A, B illustrates the findings from the roughness tests. No significant difference was found in Ra and Rz values between the As-built and HT groups. However, a significant difference was found between the EFSF group and the other two groups (*p* < 0.01).

The results concerning SFE values and wettability are detailed in Figures 7C–E. The SFE and water contact angle values showed significant differences among the three groups (all *p* < 0.01 except *p* < 0.05 between the As-built and HT groups of the water contact angle). The EFSF group presented the highest SFE value and lowest water contact angle, affirming increased hydrophilicity, followed by the HT group. In contrast, the As-built group showed the lowest SFE value and highest water contact angle.

### 3.4 Analysis of bacterial adhesion experiments with different treatments

**FIGURE 8:**
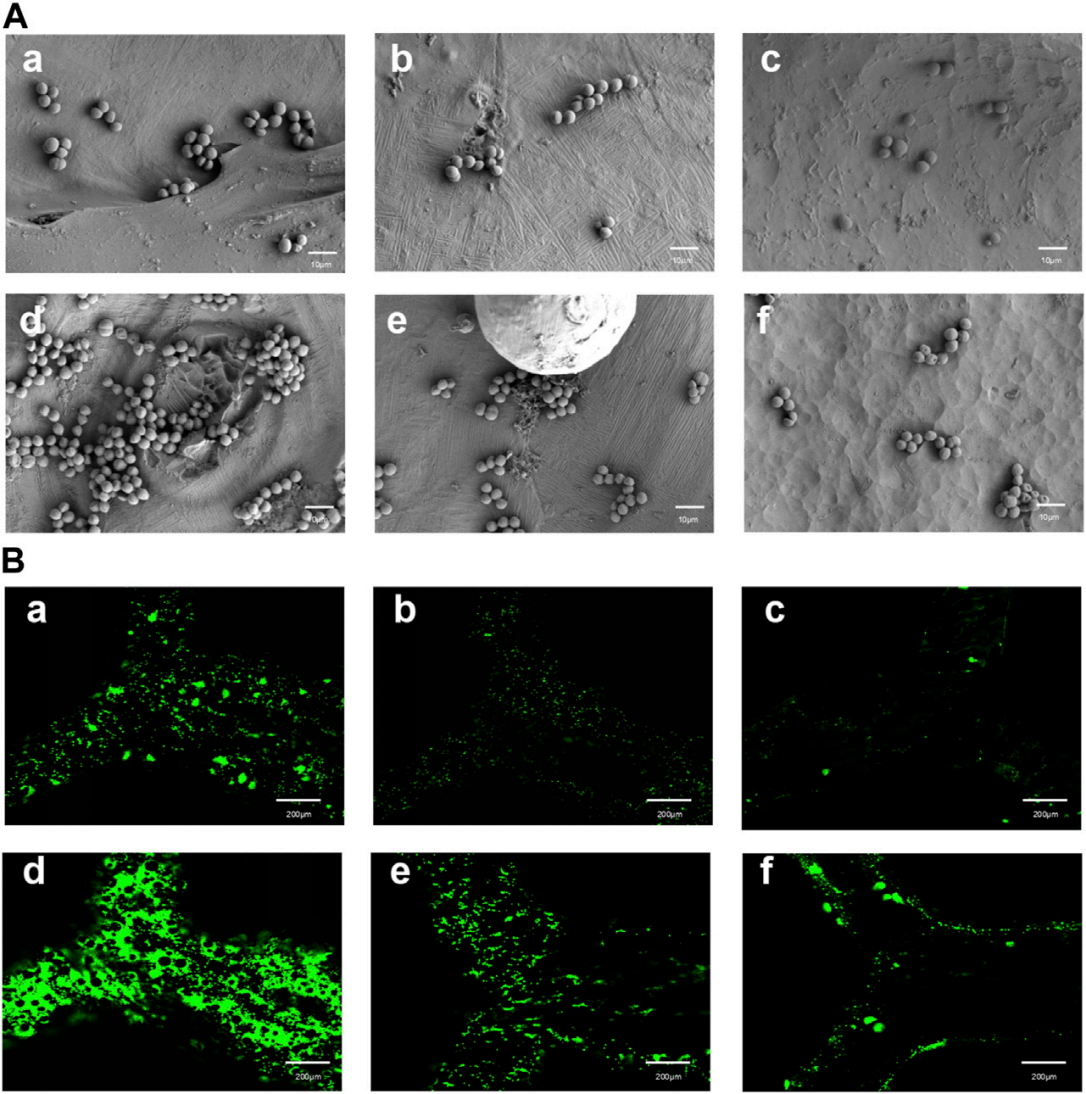
SEM and fluorescence microscopy images of TA1 specimens after specific cultivation periods and treatments. **(A) (a–f)** SEM images at a magnification of 5000× showing the surfaces of TA1 specimens after 2 h **(a–c)** and 4 h **(d–f)** of cultivation, respectively, for the printed As-built, HT, and EFSF groups. **(B) (a–f)** Fluorescence microscopy images of bacteria stained with Srty after 2 h **(a–c)** and 4 h **(d–f)** of cultivation on the surfaces of TA1 specimens, respectively, for the printed As-built, HT, and EFSF groups.

In the visualization of Sa on different surfaces, SEM images were obtained after culturing for 2 and 4 h on the surfaces of the specimens in the As-built, HT, and EFSF groups (Figure 8A). SEM observations revealed that bacteria were more prone to adhere to rough surfaces. The surface of the specimens in the HT group showed a significant reduction in bacterial adhesion; further reduction was observed in the EFSF group. The EFSF group exhibited scattered distribution of Sa bacteria with occasional small bacterial clusters. In contrast, the As-built group showed a large number of bacteria with mutual aggregation, forming clustered bacterial colonies. The HT group also exhibited clustered bacterial colonies attached to irregular pits on the surface of the specimens, but with a noticeable reduction compared with the As-built group.

Fluorescence confocal laser scanning microscopy images in Figure 8B depicts the bacterial colonies formed on the surfaces of specimens in the As-built, HT, and EFSF groups after 2 and 4 h of cultivation. These images showed that the surfaces in the HT and EFSF groups had fewer and smaller bacterial colonies compared with the surfaces in the As-built group. In particular, the smallest number of bacteria were observed on the surface of specimens in the EFSF group compared with the other two groups.

**FIGURE 9:**
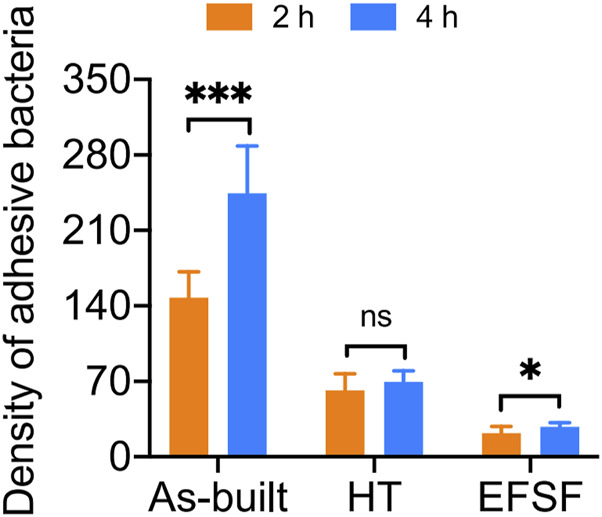
Analysis of Sa adhesion density on the surfaces of titanium scaffolds treated by three different methods.

Figure 9 presents the bacterial colony counts obtained using Image-Pro software. One-way ANOVA with Tukey’s *post hoc* test indicated a significant difference between the As-built and HT groups (*p* < 0.05), as well as between the HT and EFSF groups (*p* < 0.05).

### 3.5 Cell compatibility analysis of different treatments

**FIGURE 10:**
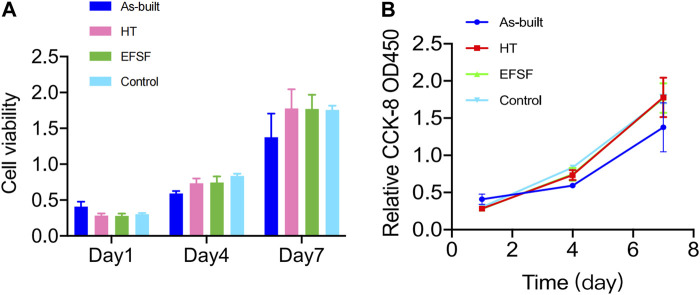
Viability of MC3T3-E1 cells measured by CCK-8 assay at different time points in each group.

Figure 10A presents the proliferation histograms of MC3T3-E1 cells, obtained from the CCK-8 assay after incubation for 1, 4, and 7 days in the four groups: the As-built, HT, and EFSF groups, along with a control group (cultured on blank plates without meshes). As the incubation time increased, the cells in each group exhibited different proliferation rates. Both the HT and EFSF groups notably surpassed the cell proliferation in the As-built group, with HT exhibiting the most pronounced growth. Although the cell proliferation in the EFSF group lagged behind that in the HT group, it demonstrated significant potential to show a marked increase (Figure 10B). The optical density values, measured using an ELISA reader at a wavelength of 450 nm, gradually increased over time, indicating remarkable cell proliferation on the meshes. The relative growth rates (RGRs) of the cells and the corresponding levels of cytotoxicity are listed in Figure 10. The RGRs in the As-built, HT, and EFSF titanium mesh groups on days 1, 4, and 7 were all above 80% compared with those in the control group, indicating nontoxicity and a cytotoxicity level of 0.

## 4 Discussion

This study investigated the mechanical properties of additively manufactured pure titanium meshes designed for oral bone augmentation. Based on prior experiments, we assessed the impact of a specific vacuum HT temperature on the mesh’s features. This treatment significantly altered the metallographic composition of the titanium mesh, resulting in grain fusion, heightened tensile strength, enhanced fracture elongation, and reinforced bending strength. Notably, the heat-treated mesh achieved a tensile force of 314.0 N and elongation of 10.56%, compared with the measurements of 297.3 N and 4.59% in the As-built group. Remarkably, the mechanical properties of the treated meshes surpassed those of the commonly used Ti-6Al-4V (TC4) alloy (Li et al., 2022), highlighting their superior potential for clinical applications. This HT method aligned with Cheng-Lin Li’s findings (Li et al., 2021), revealing that martensite structures transitioned into equiaxed grain structures at 650°C, optimizing the mesh’s microstructure. This refined structure enhanced the mechanical reliability of the mesh, rendering it suitable for demanding clinical scenarios. The robust tensile and bending strengths evident in both heat-treated and As-built samples further validated the effectiveness of the manufacturing process.

The fracture elongation of the heat-treated samples significantly increased, with the HT group showing an improvement of approximately 130% compared with the As-built group without negatively affecting the strength. These findings were consistent with study research suggesting that HT could refine grains, enhance fusion, increase the elongation rate, and improve the mechanical strength of the additive manufactured metals (Hu et al., 2022; Zhang et al., 2021).

SLM manufacturing leads to a high degree of surface roughness. While this can be beneficial for porous titanium implants that aim to bond with adjacent bone (Tsukanaka et al., 2016; Trevisan et al., 2018), it is less ideal for temporary implants, such as titanium mesh. For these implants, a smoother surface is required to avoid tissue irritation and unwanted bone integration (Albrektsson and Wennerberg, 2004). This study harnessed the potential of an innovative technique known as EFSF to create such a smoother surface. This method ingeniously combined plasma polishing with micro-nano bubble polishing. By meticulously managing the polishing parameters and fluid dynamics, the surface roughness of the pure titanium mesh was significantly reduced by eliminating a majority of surface defects, removing the oxide layers, and eradicating residual titanium powder particles. The Ra and Rz values of the EFSF-treated surface were 0.37 ± 0.11 and 2.46 ± 0.80 μm, respectively, signifying a smooth and refined surface ideal for the application.

This study showed that the EFSF technique not only reduced the surface roughness but also increased the hydrophilicity and SFE of the titanium mesh. The smoother surfaces with higher SFE could better resist bacterial adhesion and biofilm formation compared with rougher, lower-SFE titanium surfaces (Puckett et al., 2010; Zhang et al., 2015). Bacterial adhesion is a complex process influenced by various surface features, including morphology, phase, and surface roughness. This study showed that the as-printed surfaces typically had a higher degree of roughness, which could potentially promote bacterial adhesion. This was primarily due to the fact that rough surfaces provided a more conducive environment for biofilm deposition compared with smoother surfaces (Scheuerman et al., 1998; Katsikogianni and Missirlis, 2004). Indeed, previous studies demonstrated that increased surface roughness expanded the available area for bacterial adhesion (Gharechahi et al., 2012), enabling a greater contact surface for bacteria and facilitating stronger adhesion forces and binding capabilities (Ahn et al., 2009; Almaguer-Flores et al., 2010). Moreover, in oral clinical scenarios where the titanium mesh comes into contact with the oral environment, bacteria adhering to irregular surfaces are more likely to survive for extended periods because they are shielded from natural clearance forces and oral hygiene measures. This study observed a noteworthy reduction in bacterial adhesion on the EFSF-polished titanium mesh surface. This was an encouraging finding, considering that bacterial adhesion, particularly of strains such as Sa, was a leading cause of implant-associated infections.

However, SEM observation revealed that deep scratches, defects, and uneven roughness could not be completely removed with the EFSF technique. These surface imperfections appeared to be predominantly induced by fluctuations and changes in SLM process parameters, external environment, and melt pool state (Wang, 2014; Yadollahi and Shamsaei, 2017). For titanium meshes with significant defects, it seems necessary to apply preliminary treatments, such as mechanical polishing, to the rough surface prior to EFSF processing to attain a less coarse finish. Alternatively, the EFSF may be worth considering as an intermediate polishing procedure, necessitating further exploration into its potential compatibility and combination with other polishing techniques in subsequent stages.

One potential concern associated with electrochemical plasma polishing pertains to the enrichment of cytotoxic vanadium elements on the surfaces of titanium alloy devices. However, this study demonstrated that the plasma electrolytic polishing of pure titanium, due to its single-element composition, did not adversely affect cell lifespan (Bernhardt et al., 2021). Furthermore, this study found no significant difference in cell death rates for specimens in the HT and EFSF groups, indicating their good biocompatibility (Figure 10).

Despite the promising results, further research should be conducted to refine these improvement methods. The crucial next steps are exploring different polishing parameters and conducting comprehensive biocompatibility assessments, including *in vivo* implantation and cytotoxicity testing. Additionally, long-term clinical observations and studies are necessary to ascertain the effectiveness and safety of these improvement techniques in practical oral implant surgeries.

## 5 Conclusion

This study illuminated the advantageous impact of both HT and EFSF polishing on titanium mesh samples, crafted using additive manufacturing with pure titanium (TA1). The application of HT at 700°C for 90 min significantly bolstered the mechanical properties of the mesh, whereas the use of EFSF processing effectively mitigated surface roughness. Importantly, both these treatments demonstrate biocompatibility, as they show no deleterious effects on osteogenic cells. Additionally, these treatments have the potential to improve resistance against bacterial adhesion, which is a crucial parameter for successful implant surgery.

The insights gained from this study might lay the foundation for a future where the aforementioned treatments can substantially improve the properties of titanium mesh for oral implant surgeries. However, further investigations should focus on assessing the long-term clinical outcomes of these treatment methods.

## Data Availability

The original contributions presented in the study are included in the article/Supplementary material, further inquiries can be directed to the corresponding authors.

## References

[B1] Ahn H. B. Ahn S. J. Lee S. J. Kim T. W. Nahm D. S. (2009). Analysis of surface roughness and surface free energy characteristics of various orthodontic materials. Am. J. Orthod. Dentofac. Orthop. 136 (5), 668–674. 10.1016/j.ajodo.2007.11.032 19892283

[B2] Albrektsson T. Wennerberg A. (2004). Oral implant surfaces: Part 1--review focusing on topographic and chemical properties of different surfaces and *in vivo* responses to them. Int. J. Prosthodont. 17 (5), 536–543. 15543910

[B3] Almaguer-Flores A. Ximénez-Fyvie L. A. Rodil S. E. (2010). Oral bacterial adhesion on amorphous carbon and titanium films: effect of surface roughness and culture media. J. Biomed. Mat. Res. B Appl. Biomater. 92 (1), 196–204. 10.1002/jbm.b.31506 19810113

[B4] Bernhardt A. Schneider J. Schroeder A. Papadopoulous K. Lopez E. Brückner F. (2021). Surface conditioning of additively manufactured titanium implants and its influence on materials properties and *in vitro* biocompatibility. Mat. Sci. Eng. C 119, 111631. 10.1016/j.msec.2020.111631 33321670

[B5] Boyne P. J. Cole M. D. Stringer D. Shafqat J. P. (1985). A technique for osseous restoration of deficient edentulous maxillary ridges. J. Oral Maxillofac. Surg. 43 (2), 87–91. 10.1016/0278-2391(85)90054-0 3881576

[B6] Buser D. Dula K. Hirt H. P. Schenk R. K. (1996). Lateral ridge augmentation using autografts and barrier membranes: a clinical study with 40 partially edentulous patients. J. Oral Maxillofac. Surg. 54 (4), 420–432. 10.1016/s0278-2391(96)90113-5 8600258

[B7] Dank A. Aartman I. H. A. Wismeijer D. Tahmaseb A. (2019). Effect of dental implant surface roughness in patients with a history of periodontal disease: a systematic review and meta-analysis. Int. J. Implant Dent. 5 (1), 12. 10.1186/s40729-019-0156-8 30756245 PMC6372709

[B8] De Angelis N. Solimei L. Pasquale C. Alvito L. Lagazzo A. Barberis F. (2021). Mechanical properties and corrosion resistance of tial6v4 alloy produced with SLM technique and used for customized mesh in bone augmentations. Appl. Sci. 11 (12), 5622. 10.3390/app11125622

[B9] Dong Y. P. Tang J. C. Wang D. W. Wang N. He Z. D. Li J. (2020). Additive manufacturing of pure Ti with superior mechanical performance, low cost, and biocompatibility for potential replacement of Ti-6Al-4V. Mat. Des. 196, 109142. 10.1016/j.matdes.2020.109142

[B10] Gharechahi M. Moosavi H. Forghani M. (2012). Effect of surface roughness and materials composition. J. Biomater. Nanobiotechnol. 3, 541–546. 10.4236/jbnb.2012.324056

[B11] Gomes C. C. Moreira L. M. Santos V. J. Ramos A. S. Lyon J. P. Soares C. P. (2011). Assessment of the genetic risks of a metallic alloy used in medical implants. Genet. Mol. Biol. 34 (1), 116–121. 10.1590/s1415-47572010005000118 21637553 PMC3085356

[B12] Hu Y. Chen H. Jia X. Liang X. Lei J. (2022). Heat treatment of titanium manufactured by selective laser melting: Microstructure and tensile properties. J. Mat. Res. Technol. 18, 245–254. 10.1016/j.jmrt.2022.02.106

[B13] Kasemo B. Gold J. (1999). Implant surfaces and interface processes. Adv. Dent. Res. 13, 8–20. 10.1177/08959374990130011901 11276751

[B14] Katsikogianni M. Missirlis Y. F. (2004). Concise review of mechanisms of bacterial adhesion to biomaterials and of techniques used in estimating bacteria-material interactions. Eur. Cell. Mat. 8, 37–57. 10.22203/ecm.v008a05 15593018

[B15] Lee S. H. Moon J. H. Jeong C. M. Bae E. B. Park C. E. Jeon G. R. (2017). The mechanical properties and biometrical effect of 3D preformed titanium membrane for guided bone regeneration on alveolar bone defect. Biomed. Res. Int. 2017, 1–12. 10.1155/2017/7102123 PMC5605874 29018818

[B16] Li C.-L. Wang C.-S. Narayana P. L. Hong J.-K. Choi S.-W. Kim J. H. (2021). Formation of equiaxed grains in selective laser melted pure titanium during annealing. J. Mat. Res. Technol. 11, 301–311. 10.1016/j.jmrt.2021.01.008

[B17] Li L. Gao H. Wang C. Ji P. Huang Y. Wang C. (2022). Assessment of customized alveolar bone augmentation using titanium scaffolds vs polyetheretherketone (peek) scaffolds: a comparative study based on 3D printing technology. ACS Biomater. Sci. Eng. 8 (5), 2028–2039. 10.1021/acsbiomaterials.2c00060 35443132

[B18] Liu X. Chen S. Tsoi J. K. H. Matinlinna J. P. (2017). Binary titanium alloys as dental implant materials-a review. Regen. Biomater. 4 (5), 315–323. 10.1093/rb/rbx027 29026646 PMC5633690

[B19] McCracken M. (1999). Dental implant materials: commercially pure titanium and titanium alloys. J. Prosthodont. 8 (1), 40–43. 10.1111/j.1532-849x.1999.tb00006.x 10356553

[B20] Merli M. Mariotti G. Moscatelli M. Motroni A. Mazzoni A. Mazzoni S. (2015). Fence technique for localized three-dimensional bone augmentation: a technical description and case reports. Int. J. Periodontics Restor. Dent. 35 (1), 57–64. 10.11607/prd.2029 25734707

[B21] Pei G. X. Wei K. H. Jin D. (2006). Experimental technology of tissue engineering. Beijing: People’s Military Medical Press.

[B22] Puckett S. D. Taylor E. Raimondo T. Webster T. J. (2010). The relationship between the nanostructure of titanium surfaces and bacterial attachment. Biomaterials 31 (4), 706–713. 10.1016/j.biomaterials.2009.09.081 19879645

[B23] Rakhmatia Y. D. Ayukawa Y. Furuhashi A. Koyano K. (2013). Current barrier membranes: titanium mesh and other membranes for guided bone regeneration in dental applications. J. Prosthodont. Res. 57 (1), 3–14. 10.1016/j.jpor.2012.12.001 23347794

[B24] Rao S. Ushida T. Tateishi T. Okazaki Y. Asao S. (1996). Effect of Ti, Al, and V ions on the relative growth rate of fibroblasts (L929) and osteoblasts (MC3T3-E1) cells. Biomed. Mat. Eng. 6 (2), 79–86. 10.3233/bme-1996-6202 8761518

[B25] Ribeiro M. Monteiro F. J. Ferraz M. P. (2012). Infection of orthopedic implants with emphasis on bacterial adhesion process and techniques used in studying bacterial-material interactions. Biomatter 2 (4), 176–194. 10.4161/biom.22905 23507884 PMC3568104

[B26] Santhosh Kumar S. Hiremath S. S. Ramachandran B. Muthuvijayan V. (2019). Effect of surface finish on wettability and bacterial adhesion of micromachined biomaterials. Biotribology 18, 100095. 10.1016/j.biotri.2019.100095

[B27] Scarano A. Crocetta E. Quaranta A. Lorusso F. (2018). Influence of the thermal treatment to address a better osseointegration of Ti6Al4V dental implants: histological and histomorphometrical study in a rabbit model. Biomed. Res. Int. 2018, 1–7. 10.1155/2018/2349698 PMC6040305 30050922

[B28] Scheuerman T. R. Camper A. K. Hamilton M. A. (1998). Effects of substratum topography on bacterial adhesion. J. Colloid. Interface Sci. 208 (1), 23–33. 10.1006/jcis.1998.5717 9820746

[B29] Sumida T. Otawa N. Kamata Y. U. Kamakura S. Mtsushita T. Kitagaki H. (2015). Custom-made titanium devices as membranes for bone augmentation in implant treatment: clinical application and the comparison with conventional titanium mesh. J. Craniomaxillofac. Surg. 43 (10), 2183–2188. 10.1016/j.jcms.2015.10.020 26603108

[B30] Trevisan F. Calignano F. Aversa A. Marchese G. Lombardi M. Biamino S. (2018). Additive manufacturing of titanium alloys in the biomedical field: processes, properties and applications. J. Appl. Biomater. Funct. Mat. 16 (2), 57–67. 10.5301/jabfm.5000371 28967051

[B31] Tsukanaka M. Fujibayashi S. Takemoto M. Matsushita T. Kokubo T. Nakamura T. (2016). Bioactive treatment promotes osteoblast differentiation on titanium materials fabricated by selective laser melting technology. Dent. Mat. J. 35 (1), 118–125. 10.4012/dmj.2015-127 26830832

[B32] Ulrich Sommer J. Birk R. Hörmann K. Stuck B. A. (2014). Evaluation of the maximum isometric tongue force of healthy volunteers. Eur. Arch. Otorhinolaryngol. 271 (11), 3077–3084. 10.1007/s00405-014-3103-6 24970288

[B33] Wang H. (2014). Materials’ fundamental issues of laser additive manufacturing for high-performance large metallic components. Acta Aeronaut. Astronaut. Sin. 35 (10), 2690–2698. 10.7527/S1000-6893.2014.0174

[B34] Wang P. Nai M. L. S. Tan X. Vastola G. Srinivasan R. Sin W. J. (2016). “Recent progress ofAdditive manufactured Ti-6Al-4V by electron beam melting,” in Proceedings of the 2016 Annual International Solid Freeform Fabrication Symposium (SFF Symp 2016) Austin, TX, USA, 691–704.

[B35] Wang W. Xu X. Ma R. Xu G. Liu W. Xing F. (2020). The influence of heat treatment temperature on microstructures and mechanical properties of titanium alloy fabricated by laser melting deposition. Mater. (Basel) 13 (18), 4087. 10.3390/ma13184087 PMC7560422 32942530

[B36] Yadollahi A. Shamsaei N. (2017). Additive manufacturing of fatigue resistant materials: challenges and opportunities. Int. J. Fatigue 98, 14–31. 10.1016/j.ijfatigue.2017.01.001

[B37] Yang G. Wang B. Tawfiq K. Wei H. Zhou S. Chen G. (2016). Electropolishing of surfaces: theory and applications. Surf. Eng. 33 (2), 149–166. 10.1080/02670844.2016.1198452

[B38] Zeidler H. Boettger-Hiller F. Edelmann J. Schubert A. (2016). Surface finish machining of medical parts using plasma electrolytic polishing. Proc. CIRP 49, 83–87. 10.1016/j.procir.2015.07.038

[B39] Zhang J. Liu Y. Bayat M. Tan Q. Yin Y. Fan Z. (2021). Achieving high ductility in a selectively laser melted commercial pure-titanium via *in-situ* grain refinement. Scr. Mat. 191, 155–160. 10.1016/j.scriptamat.2020.09.023

[B40] Zhang X. Zhang Q. Yan T. Jiang Z. Zhang X. Zuo Y. Y. (2015). Quantitatively predicting bacterial adhesion using surface free energy determined with a spectrophotometric method. Environ. Sci. Technol. 49 (10), 6164–6171. 10.1021/es5050425 25898026 PMC4854535

